# The clinical potential of GDF15 as a “ready-to-feed indicator” for critically ill adults

**DOI:** 10.1186/s13054-020-03254-1

**Published:** 2020-09-14

**Authors:** Lisa Van Dyck, Jan Gunst, Michaël P. Casaer, Bram Peeters, Inge Derese, Pieter J. Wouters, Francis de Zegher, Ilse Vanhorebeek, Greet Van den Berghe

**Affiliations:** Clinical Division and Laboratory of Intensive Care Medicine, Department of Cellular and Molecular Medicine, KU Leuven, Herestraat 49, B-3000 Leuven, Belgium

**Keywords:** GDF15, Critical illness, Parenteral nutrition, Feeding intolerance, Outcome

## Abstract

**Background:**

Circulating growth-differentiation factor-15 (GDF15), a cellular stress marker, abruptly increases during critical illness, but its later time course remains unclear. GDF15 physiologically controls oral intake by driving aversive responses to nutrition. Early parenteral nutrition (PN) in ICU patients has overall been shown not beneficial. We hypothesized that low GDF15 can identify patients who benefit from early PN, tolerate enteral nutrition (EN), and resume spontaneous oral intake.

**Methods:**

In secondary analyses of the EPaNIC-RCT on timing of PN initiation (early PN versus late PN) and the prospective observational DAS study, we documented the time course of circulating GDF15 in ICU (*N* = 1128) and 1 week post-ICU (*N* = 72), compared with healthy subjects (*N* = 65), and the impact hereon of randomization to early PN versus late PN in propensity score-matched groups (*N* = 564/group). Interaction between upon-admission GDF15 and randomization for its outcome effects was investigated (*N* = 4393). Finally, association between GDF15 and EN tolerance in ICU (*N* = 1383) and oral intake beyond ICU discharge (*N* = 72) was studied.

**Results:**

GDF15 was elevated throughout ICU stay, similarly in early PN and late PN patients, and remained high beyond ICU discharge (*p* < 0.0001). Upon-admission GDF15 did not interact with randomization to early PN versus late PN for its outcome effects, but higher GDF15 independently related to worse outcomes (*p* ≤ 0.002). Lower GDF15 was only weakly related to gastrointestinal tolerance (*p* < 0.0001) and a steeper drop in GDF15 with more oral intake after ICU discharge (*p* = 0.05).

**Conclusion:**

In critically ill patients, high GDF15 reflected poor prognosis and may contribute to aversive responses to nutrition. However, the potential of GDF15 as “ready-to-feed indicator” appears limited.

**Trial registration:**

ClinicalTrials.gov, NCT00512122, registered 31 July 2007, https://www.clinicaltrials.gov/ct2/show/NCT00512122 (EPaNIC trial) and ISRCTN, ISRCTN 98806770, registered 11 November 2014, http://www.isrctn.com/ISRCTN98806770 (DAS trial)

## Background

Several large RCTs have investigated the use of enhanced artificial nutrition during the early course of critical illness [1]. When combining all evidence, early supplemental parenteral or enhanced enteral feeding does not appear to benefit and may even harm critically ill patients. However, it is currently unknown how to identify the best time to initiate supplemental parenteral or enhanced enteral feeding for an individual patient [1]. Indeed, varying study results, possibly explained by different study designs and patient populations, did not allow to unequivocally recommend on the best timing, which thus remains at the discretion of the treating physician [2]. It can be inferred that the best timing for initiating enhanced artificial nutrition, comprising enhanced enteral and/or supplemental parenteral nutrition, depends on the phase of the recovery process and varies per patient. Thus, it would be of great value to have a sensitive and specific biomarker that identifies, at individual patient level, the time at which enhanced artificial nutrition will be effectively used for anabolism and recovery. Growth-differentiation factor-15 (GDF15) could be a promising candidate.

GDF15 is a stress response cytokine that is induced by injury and inflammation [3, 4]. Hence, it is not surprising that in critically ill patients, very high circulating GDF15 levels have been observed, which were associated with risk of death in a few small studies [5–8]. Also mitochondrial dysfunction, high-fat feeding, and unbalanced protein underfeeding have been shown to increase circulating GDF15 levels [3, 9]. Recent studies in animal models and other settings have revealed that GDF15 drives anorexia, through induction of nausea, vomiting, and an aversive reaction to food [9–11]. Unsurprisingly, elevated circulating GDF15 levels have been shown to be involved in anorexia nervosa, cancer cachexia, and obesity [12]. These findings suggest that GDF15 may also be a driver of critical illness-induced anorexia and gastrointestinal intolerance [13, 14].

In other settings like obesity, induction of GDF15 has been considered a physiological signal of reduced nutritional need and cellular inability to metabolize macronutrients and as such to aid in restoring homeostasis [12]. Inferentially, elevated GDF15 could function as signal to withhold nutrition in an individual critically ill patient at a time when it cannot be used for anabolism, or vice versa, low GDF15 could identify a good time to start refeeding. Hitherto, the time course of serum GDF15 levels during critical illness and recovery has not been well documented, with only one study reporting values the first three days after cardiogenic shock [8]. Also, the relation between GDF15 and nutrition during critical illness has not been investigated. We hypothesized that low GDF15 can identify patients who benefit from early parenteral nutrition (PN), who tolerate enteral nutrition (EN), and who resume spontaneous oral intake.

## Methods

### Study design

This is a secondary analysis of the randomized controlled “EPaNIC” trial (ClinicalTrials.gov-NCT00512122) and the prospective observational “DAS” trial (ISRCTN98806770). The EPaNIC trial investigated the impact of withholding PN during the first week in ICU (late PN) versus early initiation of supplemental PN (early PN) in a heterogeneous cohort of 4640 adult critically ill patients [15, 16]. Patients randomized to early PN received supplemental PN within 48 h after ICU admission when EN alone was insufficient to reach caloric targets. In patients randomized to late PN, initiation of PN was postponed until day 8 in ICU, whereby a macronutrient deficit resulting from EN intolerance was accepted. Late PN patients received intravenous 5% dextrose to provide similar adequate hydration as early PN patients. All patients received EN as soon as possible, parenteral trace elements, minerals and vitamins, and insulin infusions targeting normoglycemia (80–110 mg/dL). Blood samples were obtained via an arterial catheter upon ICU admission and daily at 06:00 ± 2 h, and serum was stored at − 80 °C. The detailed protocol and primary outcomes have been published [15, 16]. In contrast with the EPaNIC trial, in which both short- and long-stay patients were included, the DAS trial only included patients who stayed in ICU for at least 7 days and were still dependent on vital organ support (defined as need for mechanical ventilation, and mechanical and/or pharmacological hemodynamic support) at that time. Blood samples were obtained at 6:00 ± 2 h via an arterial catheter, daily from day 7 until day 28 in ICU, then weekly until ICU discharge, at ICU discharge, and 7 days post-ICU if patients were still admitted to a regular hospital ward. Plasma was stored at − 80 °C. Patients were fed like the late PN group in EPaNIC. Primary outcomes have been published [17]. More detailed inclusion and exclusion criteria for both studies are listed in Additional table 1. The Ethical Committee Research UZ/KU Leuven approved both study protocols (ML4190 and S57249). Written informed consent was obtained from all patients or their next of kin.

### Patient selection

To investigate the GDF15 response to critical illness and the impact hereon of early PN versus late PN, we quantified GDF15 serum/plasma concentrations at different time points during and after critical illness. The in-ICU GDF15 time course was investigated for EPaNIC-patients; GDF15 recovery post-ICU was investigated for DAS-patients.

When planning this study, given the lack of prior data on in-ICU dynamics of GDF15, it was not possible to estimate an effect size and calculate a sample size. For logistic reasons, we quantified GDF15 over time in relation to randomization and survival status in a previously selected subset of patients (*n* = 1128) with available serum samples upon admission and the last ICU day and on days 4 and 7 if still in ICU at that time [18]. To avoid selection bias due to non-random missing of samples, 564 early PN and 564 late PN patients were propensity score-matched for baseline characteristics. Logistic regression was used to estimate propensity scores based on age, gender, body mass index (BMI), severity (Acute Physiology and Chronic Health Evaluation II score or APACHE-II score) and type of illness, risk of malnutrition (nutritional risk screening score or NRS score [19]), history of malignancy, diabetes, pre-inclusion dialysis, sepsis upon admission, and emergency versus elective admission. One-to-one nearest neighbor matching with greedy matching algorithm without replacement and caliper 0.00025 was used. A matched subgroup of these patients with ICU stay of at least 4 days was used for additional GDF15 quantification on days 1, 2, and 3. Propensity score matching with caliper 0.015 yielded a selection of 153 early PN and 153 late PN patients. To document GDF15 recovery, all DAS study patients were included with available plasma samples at the last ICU day and 7 days after ICU discharge, as well as data on oral nutrient intake during the first week post-ICU (*n* = 72). Sixty-five healthy control subjects, with age, gender, and BMI comparable to the patients, were included as healthy reference. Control serum samples were taken in fed condition during a preoperative consultation prior to elective minor surgery.

To identify patient characteristics independently associated with upon ICU admission GDF15 concentrations, and to investigate whether upon admission GDF15 was determining benefit or harm from early PN versus late PN, GDF15 concentrations were quantified in all available ICU admission serum samples of the EPaNIC trial (*n* = 4393). To investigate the association of GDF15 with gastrointestinal tolerance of enteral and oral nutrition, serum GDF15 concentrations were quantified in all available ICU day 4 samples of EPaNIC-patients who stayed at least 7 days in ICU (*n* = 1383). This selection was based on the assumption that for long-stay patients, at least one attempt to start EN should have been made by day 4 in ICU, while excluding patients who did not receive EN due to expected shorter stay. The association between GDF15 recovery post-ICU and oral nutrient intake was investigated for the 72 DAS study patients described above.

**Fig. 1:**
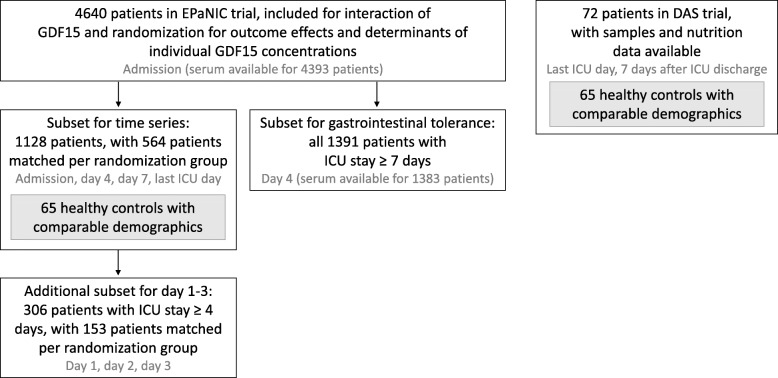
Patient selection. Studied samples are depicted in gray. Randomization group refers to early PN versus late PN. GDF15, growth-differentiation factor-15; ICU, intensive care unit

A schematic presentation of the study participants is shown in Fig. 1. Baseline characteristics and main outcomes of all groups are described in Table 1. Energy intake for matched early PN and late PN patients is shown in Additional Figure 1.

**Table 1:** Baseline characteristics and main outcomes for patients and controls

	Controls	Included EPaNIC RCT patients				Propensity score-matched sub-cohort for impact of randomization on time series			Propensity score-matched sub-cohort for impact of randomization on day 1,2,3			Included DAS trial patients
		Admission sample available	Patients in time course study	Patients with MRC sum score	Patients in GI tolerance study	Late PN	Early PN		Late PN	Early PN		
	*N* = 65	*N* = 4393	*N* = 1128	*N* = 600	*N* = 1379	*N* = 564	*N* = 564	*P*	*N* = 153	*N* = 153	*P*	*N* = 72
**Baseline characteristics**												
Gender—male, *n* (%)	41 (63.1)	2827 (64.4)	751 (66.6)	353 (58.8)	865 (62.7)	371 (65.8)	380 (67.4)	0.57	96 (62.8)	100 (65.4)	0.63	41 (56.9)
Age—years, median [IQR]	67 [56–75]	66 [56–75]	66 [57–74]	63 [53–73]	65 [54–75]	68 [57–75]	66 [55–74]	0.18	69 [59–77]	69 [58–76]	0.41	62 [46–75]
BMI—kg/m^2^, median [IQR]	26 [23–28]	26 [23–29]	26 [23–28]	25 [23–29]	25 [23–29]	26 [24–28]	26 [23–28]	0.25	25 [23–28]	25 [22–28]	0.61	25 [23–29]
Diabetes mellitus, *n* (%)	6 (9.2)	746 (17.2)	176 (15.6)	96 (16.0)	234 (17.0)	81 (14.4)	95 (16.8)	0.25	25 (16.3)	21 (13.7)	0.52	8 (11.1)
Malignancy, *n* (%)	11 (16.9)	814 (18.8)	173 (15.3)	163 (0.27)	327 (23.7)	84 (14.9)	89 (15.8)	0.68	26 (17.0)	26 (17.0)	> 0.99	10 (13.9)
NRS score ≥ 5, *n* (%)	NA	761 (17.6)	168 (14.9)	177 (29.5)	422 (30.6)	76 (13.5)	92 (16.3)	0.18	33 (21.6)	28 (18.3)	0.47	NA
APACHE-II score, median [IQR]	NA	19 [14–31]	18 [13–29]	31 [20–37]	32 [23–37]	18 [13–28]	17 [14–29]	0.57	25 [16–34]	24 [16–33]	0.94	30 [26–36]
Pre-admission dialysis, *n* (%)	NA	62 (1.4)	10 (0.9)	6 (1.0)	25 (1.8)	4 (0.7)	6 (1.1)	0.52	1 (0.7)	2 (1.3)	0.56	NA
Sepsis upon admission, *n* (%)	NA	867 (20.0)	172 (15.3)	264 (44.0)	636 (46.1)	85 (15.1)	87 (15.4)	0.86	48 (31.4)	43 (28.1)	0.53	NA
Emergency admission, *n* (%)	NA	1701 (39.3)	367 (32.5)	413 (68.8)	1014 (73.5)	177 (31.4)	190 (33.7)	0.40	72 (47.1)	76 (49.7)	0.64	66 (91.7)
**Outcomes**												
ICU mortality, *n* (%)	NA	227 (5.2)	49 (4.3)	23 (3.8)	198 (14.4)	20 (3.6)	29 (5.1)	0.18	8 (5.2)	11 (7.2)	0.47	0 (0.0)
ICU stay days, median [IQR]	NA	3 [2–8]	3 [2–7]	12 [4–21]	14 [10–24]	3 [2–6]	3 [2–7]	0.05	7 [5–15]	7 [4–15]	0.50	15 [10–22]
New infection, *n* (%)	NA	1041 (24.0)	237 (21.0)	309 (51.5)	954 (69.2)	101 (17.9)	136 (24.1)	0.01	66 (43.1)	70 (45.8)	0.64	NA
Muscle weakness, *n* (%)	NA	218 (37.6)	38 (28.8)	232 (38.7)	226 (55.0)	NA	NA		NA	NA		NA
Respiratory support, *n* (%)	NA	4155 (96.0)	1092 (96.8)	578 (96.3)	1352 (98.0)	544 (96.5)	548 (97.2)	0.49	148 (96.7)	150 (98.0)	0.47	NA
Hemodynamic support, *n* (%)	NA	3611 (83.4)	957 (84.8)	515 (85.8)	1259 (91.3)	475 (84.2)	482 (85.5)	0.56	141 (92.2)	142 (92.8)	0.82	NA

*Abbreviations*: *MRC* Medical Research Council, *GI* gastrointestinal, *IQR* interquartile range, *BMI* body mass index, *NA* not applicable or information not available, *NRS* nutritional risk screening score, *APACHE* acute physiology and chronic health evaluation, *ICU* intensive care unit

### Serum/plasma analyses

Serum/plasma GDF15 concentrations were measured with a commercially available enzyme-linked immunosorbent assay (Human GDF15 Quantikine ELISA, R&D Systems).

### Statistical analyses

Variables were summarized as frequencies and percentages or medians and interquartile ranges (IQR), unless indicated otherwise. Baseline characteristics were compared with chi-square tests or Mann-Whitney *U* tests as appropriate. For the time course analyses, groups were compared with Mann-Whitney *U* tests at each time point, and only for patients who stayed at least 4 days in ICU additionally with repeated measures MANOVA after logarithmically transforming the data to obtain a near-normal distribution. GDF15 recovery post-ICU was investigated with repeated measures ANOVA after log transformation to obtain a near-normal distribution. Variables independently associated with upon-admission GDF15 concentrations were identified using multivariable linear regression analysis and added to the model based on literature and data availability.

To investigate whether upon-admission GDF15 was determining benefit or harm from early PN versus late PN, the interaction between GDF15 and randomization to early PN versus late PN for its outcome effects was studied stepwise, with multivariable nominal logistic regression and Cox proportional hazard analyses. First, independent associations of GDF15 and of early PN versus late PN with outcome were investigated, adjusting for five baseline risk factors: age, BMI (dichotomized as 25–40 kg/m^2^ versus other), severity of illness (APACHE-II score), simplified diagnostic category (cardiac surgery, emergency surgical, elective surgical or medical), and risk of malnutrition (NRS score, dichotomized as ≥ 5 versus < 5). Studied outcomes included time to live discharge from ICU (with censoring of patients who died beyond the longest-staying survivor), risk of acquiring a new infection in ICU, and risk of developing ICU-acquired weakness (determined in a subgroup of 600 patients and defined as a Medical Research Council Sum Score below 48 [20]). Next, both randomization to early PN versus late PN and GDF15 and their interaction were added to the models, with a significant interaction prospectively defined as interaction *p* value ≤0.15.

The association of GDF15 with tolerance of enteral/oral nutrition was investigated with logistic regression analysis, and the association with total as well as enteral and oral caloric intake in the next 24 h was investigated with linear regression. Univariable associations were subsequently adjusted for the baseline risk factors described above. Gastrointestinal tolerance of enteral/oral nutrition was defined as fulfilling at least one of the following criteria: enteral/oral nutrition successfully initiated or increased compared to the previous day, at least 70% of the caloric target met via enteral/oral nutrition, or at least 100 kcal consumed through oral intake. During the first week post-ICU, oral nutrient intake was scored semi-quantitatively over this entire week (low, moderate, and high intake corresponding to an estimated intake of less than 40%, 40–60%, and more than 60% of caloric target, respectively) and added as covariate to the repeated-measures ANOVA analysis described above. To investigate the predictive potential of GDF15 for volitional oral nutrient intake, we additionally investigated the association between GDF15 on the last day in ICU and oral intake in the first week post-ICU using a Mann-Whitney *U* test.

Statistical analyses were performed with JMP®Pro14.0.0 (SAS Institute, Cary, NC). For propensity score matching, SPSS R-menu R3.1 (Foundation for Statistical Computing) in IBM SPSS Statistics 23.0.0.0 (SPSS, Chicago, IL) was used. Two-sided *p* values ≤ 0.05 were considered to indicate statistical significance. For time course analyses, *p* values were multiplied by the number of additional time points assessed, to correct for multiple comparisons.

## Results

### Determinants of upon-admission serum GDF15 concentrations, time course of the GDF15 response to critical illness and impact hereon of early PN versus late PN

Upon ICU admission, serum GDF15 concentrations were 4.8-fold elevated (median [IQR] 4252 [2161; 8128] pg/ml) compared with healthy controls (890 [609; 1328] pg/ml, *p* < 0.0001). Variables independently associated with higher upon-admission serum GDF15 concentrations included a history of diabetes or malignancy, admission diagnosis, and higher APACHE-II scores, sepsis, corticosteroid treatment, higher plasma creatinine and total bilirubin, and lower plasma C-reactive protein (CRP) concentrations upon-admission (*R*^2^ = 0.23, Table 2).

**Table 2:** Variables associating with individual GDF15 concentrations in multivariable linear regression analysis

Model with variables explaining serum GDF15 concentrations upon ICU admission	Scaled estimate (95%CI)	***p*** value
**ICU-independent variables**		
Age	353 (− 528; 1234)	0.43
BMI	− 674 (− 2057; 709)	0.33
History of diabetes mellitus	731 (314; 1147)	0.0006
History of malignancy	485 (60; 909)	0.02
NRS score of ≥ 5 vs < 5	424 (− 24; 872)	0.06
**ICU-related variables**		
Admission diagnosis		
Cardiac surgery	16 (− 761; 793)	0.96
Elective other surgery	1566 (445; 2687)	0.006
Emergency other surgery	− 1256 (− 2013; − 499)	0.001
Medical disease	− 326 (− 1477; 824)	0.57
APACHE-II score	4454 (3211; 5697)	< 0.0001
Sepsis upon admission	2232 (594; 3869)	0.007
Infection upon admission	− 1412 (− 3059; 234)	0.09
Mechanical ventilation on ICU day 1	296 (− 452; 1044)	0.43
Steroids on ICU day 1	703 (212; 1194)	0.005
**Laboratory parameters**		
Blood glucose upon ICU admission	− 1975 (− 4405; 455)	0.11
Plasma creatinine on ICU day 1	22,102 (19,679; 24,526)	< 0.0001
Plasma total bilirubin on ICU day 1	13,569 (10,097; 17,041)	< 0.0001
Plasma CRP on ICU day 1	− 1605 (− 3132; − 78)	0.03

Scaled estimates are shown for all variables included in the linear regression model built to explain individual GDF15 concentrations upon ICU admission. Age, BMI, APACHE-II score, and the laboratory parameters were added as continuous variables; all other variables were added as categorical variables. The *R*^2^ of the model was 0.23. A sensitivity analysis using BMI (< 18.5 kg/m^2^, 18.5–25 kg/m^2^, > 25 kg/m^2^) and age (18– < 30 years, 30– < 50 years, 50– < 70 years, 70– < 90 years, > 90 years) as categorical variables was performed to rule out non-significant associations in case of non-linear relationships, which also yielded insignificant associations for all categories (data not shown)

*Abbreviations*: *GDF15* growth-differentiation factor-15, *ICU* intensive care unit, *BMI* body mass index, *NRS* nutritional risk screening, *APACHE* acute physiology and chronic health evaluation, *CRP* C-reactive protein

**Fig. 2:**
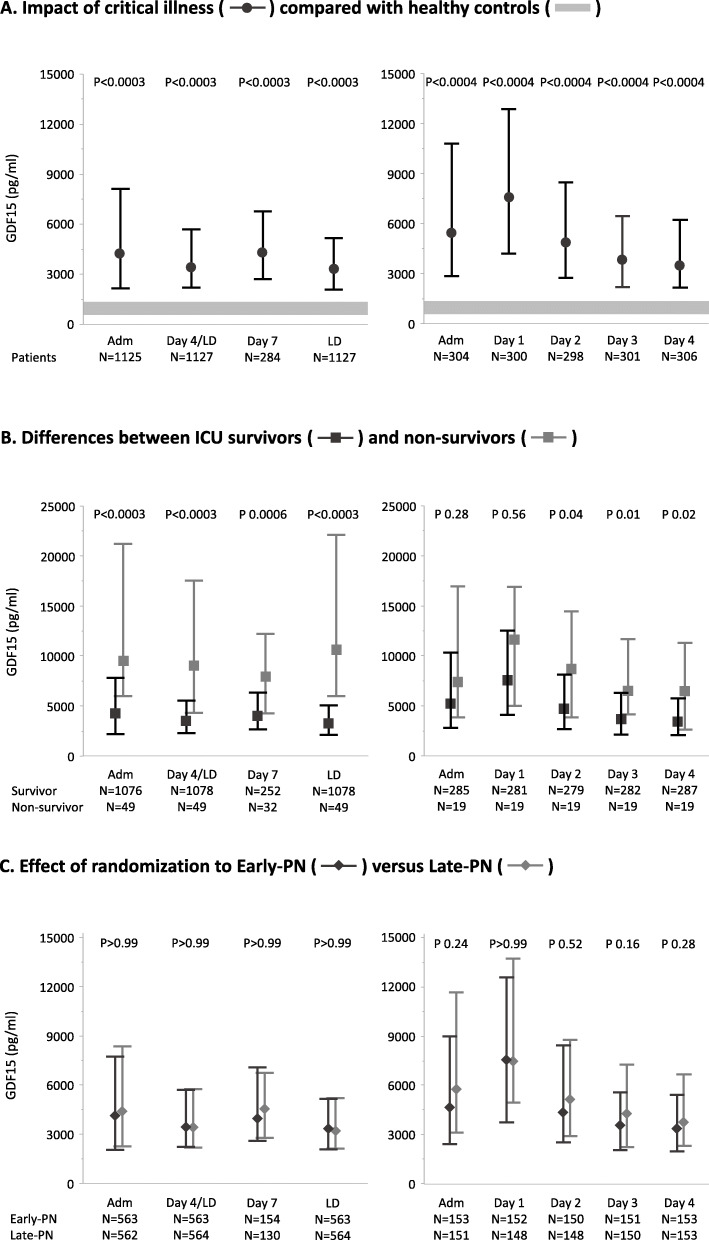
Time course of GDF15 during critical illness. Serum concentrations of GDF15 were quantified in 65 healthy controls and in 564 early PN and 564 late PN ICU patients, who were matched for upon ICU admission characteristics, on the admission day, on day 4 or the last day in ICU for patients with a shorter ICU stay (d4/LD), on day 7 for patients still in ICU on that day, and on the last ICU day (left panels). In addition, in a smaller subset of patients with an ICU stay of at least 4 days, serum GDF15 concentrations were also quantified on day 1, day 2, and day 3 in the ICU (right panels). Numbers below each graph indicate, for each time point, how many patients had sufficient serum available for GDF15 measurement and were included in the analyses. *P* values, adjusted for multiple comparisons, for each time point are shown at the top of the graphs. Geometric shapes represent medians, and whiskers represent interquartile ranges. **a** Comparison of all patients with 65 control subjects (gray area representing interquartile ranges) who had never been admitted to an ICU. **b** Comparison of patients randomized to early PN versus late PN. **c** Comparison of ICU survivors and non-survivors

Throughout critical illness, following a peak rise on ICU day 1, serum GDF15 concentrations remained elevated (*p* < 0.0001, Fig. 2a), until the last ICU day (3277 [2106; 5186] pg/ml, *p* < 0.0001). Serum GDF15 concentrations in ICU non-survivors were 2.3-fold higher (9415 [5913; 21,230] pg/ml) upon admission as compared with survivors (4161 [2108; 7764] pg/ml, *p* < 0.0001) and remained higher throughout ICU stay (all *p* ≤ 0.01 except for day 1, repeated measures MANOVA for patients with ICU stay of at least 4 days *p* = 0.07, Fig. 2b). From admission towards the last ICU day, GDF15 increased further in non-survivors (median change from baseline [IQR] 1123 [− 3912; 7314] pg/ml) and decreased in survivors (− 555 [− 3786; 1060] pg/ml, *p* = 0.004). Early PN and late PN patients had similar in-ICU GDF15 concentrations (all *p* > 0.05, repeated measures MANOVA for patients with ICU stay of at least 4 days *p* = 0.69, Fig. 2c).

For patients in ICU for at least 7 days, plasma GDF15 concentrations decreased with 26.4% from the last ICU day (2662 [1646; 5441] pg/ml) to 7 days post-ICU (1958 [1342; 4184] pg/ml, *p* = 0.0001), although levels remained 2.2-fold elevated compared with healthy controls (*p* < 0.0001).

### Upon-admission GDF15 as possible determinant for benefit or harm from early PN versus late PN

Higher upon-admission serum GDF15 concentrations and randomization to early PN versus late PN were independently associated with a lower likelihood of early live ICU discharge and higher risks of acquiring a new infection or muscle weakness (Table 3). There was no statistical interaction between upon-admission GDF15 concentrations and randomization to early PN versus late PN for these outcomes (interaction *p* values > 0.15, Table 3).

**Table 3:** Interaction between serum GDF15 concentrations upon ICU admission and randomization to early PN versus late PN for impact on clinical outcome

Outcome		
**Likelihood of earlier live discharge from ICU**	**HR (95% CI)**	***p***
1A. GDF15 (per ng/ml added)	0.957 (0.947–0.967)	< 0.0001
1B. Randomization to early PN vs late PN	0.939 (0.883–0.998)	0.04
2. GDF15, randomization and interaction		
GDF15 (per ng/ml added)		< 0.0001
Randomization to early PN vs late PN		0.02
Interaction GDF15-randomization		0.21
**Risk of new infection**	**OR (95% CI)**	***p***
1A. GDF15 (per ng/ml added)	1.014 (1.007–1.021)	0.0001
1B. Randomization to early PN vs late PN	1.245 (1.071–1.447)	0.004
2. GDF15, randomization and interaction		
GDF15 (per ng/ml added)		< 0.0001
Randomization to early PN vs late PN		0.003
Interaction GDF15-randomization		0.46
**Risk of ICU acquired weakness**	**OR (95% CI)**	***p***
1A. GDF15 (per ng/ml added)	1.026 (1.006–1.045)	0.006
1B. Randomization to early PN vs late PN	1.457 (1.001–2.122)	0.04
2. GDF15, randomization and interaction		
GDF15 (per ng/ml added)		0.002
Randomization to early PN vs late PN		0.03
Interaction GDF15-randomization		0.19

Stepwise multivariable models were built including all patients with upon ICU admission serum sample available for GDF15 measurement (*N* = 4393). Step 1: Models investigating the independent effect of GDF15 concentrations upon ICU admission (step 1A) and randomization to early PN vs late PN (step 1B) on outcome. All models were adjusted for the baseline risk factors age, body mass index, severity of illness, diagnostic category (cardiac surgery, emergency surgical, elective surgical and medical ICU admission), and risk of malnutrition. Step 2: Both GDF15 concentrations upon ICU admission and randomization to early PN versus late PN, as well as the interaction between randomization and GDF15 were included in the model, adjusting for the same baseline risk factors. This allowed to investigate whether upon-admission GDF15 was determining benefit or harm from early PN versus late PN

*Abbreviations*: *ICU* intensive care unit, *OR* odds ratio, *CI* confidence interval, *GDF15* growth-differentiation factor-15, *PN* parenteral nutrition, *HR* hazard ratio

### Serum GDF15 concentrations in relation to gastrointestinal tolerance of enteral/oral nutrition

**Fig. 3:**
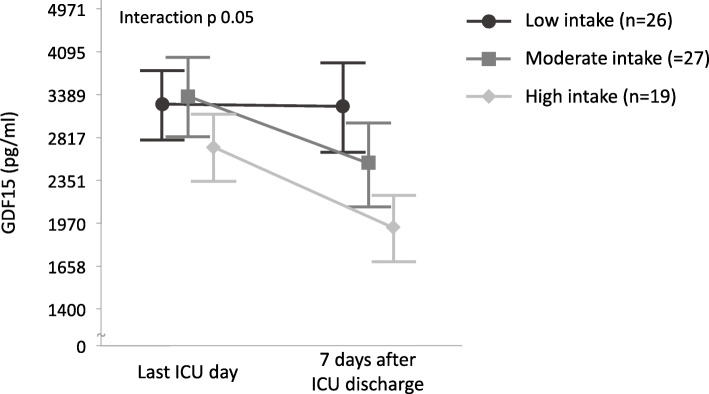
Relation of GDF15 with oral intake after ICU discharge. Plasma concentrations of GDF15 were quantified in 72 ICU patients on the last day in ICU and 7 days after ICU discharge. Macronutrient intake was scored semi-quantitatively based on estimated nutrient intake (low, moderate, and high intake meaning respectively < 40%, 40–60%, or > 60% of a normal intake). Geometric shapes represent means, and error bars represent standard errors of the mean. Numbers in the figure legend indicate the number of patients per group. Data are shown on a logarithmic scale

For patients who stayed at least 7 days in ICU, serum GDF15 concentrations on ICU day 4 were inversely but weakly associated with tolerance of enteral/oral nutrition in the following 24 h (odds ratio for risk of intolerance per ng/ml GDF15 increase: 1.027 [1.012; 1.042], *p* < 0.0001, *R*^2^ = 0.008), with limited sensitivity and specificity (area under receiver operating curve (AUROC) 0.57), and inversely but weakly associated with total as well as enteral and oral caloric intake in the following 24 h (scaled estimate [95% confidence interval] − 389 [− 714; − 64], *p* = 0.01, *R*^2^ 0.003 for total intake; − 324 [− 525; − 124], *p* = 0.001, *R*^2^ 0.007 for enteral and oral intake). These associations were independent of baseline risk factors (adjusted odds ratio per ng/ml GDF15 increase: 1.031 [1.015; 1.047], *p* < 0.0001, *R*^2^ = 0.04, AUROC = 0.64 for tolerance; adjusted scaled estimate − 514 [− 840; − 187], *p* = 0.002, *R*^2^ 0.04 for total intake; adjusted scaled estimate − 446 [− 643; − 248], *p* < 0.0001, *R*^2^ 0.08 for enteral and oral intake). During the 7 days post-ICU, patients with more nutrient intake showed a steeper GDF15 decrease (delta GDF15 median [IQR] for low intake − 79 [− 1532; 649] pg/ml, for moderate intake − 509 [− 1780; − 94] pg/ml, for high intake − 530 [− 3576; − 88] pg/ml, interaction *p* = 0.05, Fig. 3). GDF15 concentrations on the last ICU day were not related with nutrient intake in the first week post-ICU (GDF15 median [IQR] for low intake 3484 [1936–5318] pg/ml, for moderate intake 2974 [1625–7423] pg/ml, for high intake 2131 [1564–4723] pg/ml, *p* = 0.62).

## Discussion

In this secondary analysis of the EPaNIC-RCT and the observational DAS study, we demonstrated a 4.8-fold increase in serum GDF15 upon ICU admission, a rise related to higher illness severity and the presence of comorbidities. Throughout ICU stay, after a peak on day 1, GDF15 remained elevated, more so in non-survivors than in survivors. In long-stay survivors, GDF15 concentrations subsequently decreased to some extent over the first 7 days post-ICU, but were still twice the levels of healthy control subjects. Patients randomized to early PN versus late PN had similar GDF15 concentrations, and, vice versa, the upon-admission GDF15 level was not associated with benefit or harm from early PN as compared with late PN. Although lower GDF15 levels in ICU predicted to some extent better gastrointestinal tolerance, and a steeper fall in GDF15 post-ICU was associated with more oral nutrient intake, the potential of GDF15 as “ready-to-feed indicator” was limited.

Other studies have also reported elevated upon-admission GDF15 concentrations in selected, relatively small cohorts of ICU patients, which correlated with poor outcome [5–8]. However, the time course beyond the acute phase and during recovery was not investigated. We here confirmed very high upon-admission GDF15 levels in a large and heterogeneous cohort of ICU patients. We here also documented the GDF15 time course throughout ICU stay until the recovery phase post-ICU. Unexpectedly, GDF15 concentrations were found to decrease only slightly over time in ICU survivors, with a persistent and important elevation of GDF15 levels still present 7 days post-ICU. Post-ICU data, however, were only available for patients who stayed for at least 7 days in ICU, making it unclear whether a persistent elevation after ICU discharge is also present in less severely ill patients. Nevertheless, this finding suggests that cell damage may still be present in the recovery phase of critical illness. The observed rise in GDF15 concentrations in response to critical illness was also much larger than the increase that was previously reported for other pathological conditions such as obesity, and severity of illness appeared to be an important determinant [21, 22]. Therefore, the substantial GDF15 rise likely reflects the degree of cellular damage evoked by critical illness [23]. Indeed, GDF15 is known to be regulated by the integrated stress response (ISR) [3, 9], and studies in a clinically relevant animal model of critical illness have documented activation of the ISR, likely having an adaptive role [24]. In addition, pronounced inflammation and mitochondrial dysfunction may play a role [3, 4, 25]. GDF15 was unaffected by early PN versus late PN and thus the amount of macronutrients did not drive these high levels. This was somewhat unexpected given that studies in experimental models reported GDF15 alterations with nutritional interventions [9]. The lack of effect of nutrition in our study may be explained by the relatively short duration of the intervention or by the sufficiently balanced macronutrient composition of the commercial EN and PN formulas, since GDF15 increases have mainly been associated with unbalanced high-fat or low-protein diets [9]. Finally, the overwhelming response to critical illness may have precluded any additional impact of early PN versus late PN, since the reported effects on GDF15 of nutritional interventions were more moderate [9].

Research in other settings has proposed GDF15 as an important physiological signal of low metabolic needs or inability to process macronutrients [3, 10, 11], hence suggesting that low GDF15 in ICU patients may indicate when patients could benefit from early enhanced nutrition [12]. However, there was no statistical interaction between upon-admission GDF15 concentrations and early PN versus late PN for its outcome effects. Hence, these data do not support clinical potential for GDF15 as biomarker to indicate when an individual patient might metabolically tolerate parenteral nutrition.

Previous work showed that GDF15 physiologically reduces oral nutrient intake through induction of anorexia, nausea, and vomiting [9, 11, 13, 14]. Hence, the persistent GDF15 elevation in critically ill patients may mediate gastro-intestinal intolerance of enteral feeding and may contribute to their anorexia. In our study, higher GDF15 concentrations were, albeit weakly, associated with poor tolerance of in-ICU enteral/oral feeds and with low post-ICU oral intake in the recovery phase. However, the low sensitivity and specificity suggest that any potential of GDF15 as predictor of gastrointestinal feeding (in)tolerance at individual patient level is limited, which was also highlighted by the absence of a significant correlation between GDF15 on the last day in ICU and oral nutrient intake in the week after discharge.

One strength of the current study was its large sample size and the prospectively collected blood samples repeatedly over time, which allowed to characterize the GDF15 time course in a mixed patient cohort throughout and beyond ICU stay. Another strength, the randomized controlled study design of EPaNIC, allowed to reliably investigate predictive power of upon-admission GDF15 levels for benefit or harm from the early use of PN in ICU. There are also limitations to highlight. First, for the time course analysis and the impact hereon of early PN versus late PN, lack of pre-existing data precluded a priori statistical power calculation. However, a role for upon-admission GDF15 in identifying who could benefit or be harmed by early PN could be excluded, given the large sample size and the randomized controlled study design of the EPaNIC study. It does remain unclear whether GDF15 has any such discriminative potential at later time points in ICU. Furthermore, investigation of any relation between GDF15 and gastrointestinal (in)tolerance in this study was studied at selected time points and observational, as the amount of EN was not determined by randomization. Inherently, the observed associations do not prove causation, which would require interference with biological availability of GDF15. In addition, the amount of EN given in this study was rather conservative [26], which potentially underestimated the incidence of gastrointestinal intolerance. Furthermore, our definition of gastrointestinal intolerance may have been confounded by fasting for reasons not related to feeding intolerance (e.g., preprocedural fasting). Finally, macronutrient intake post-ICU was estimated based on a limited amount of available data.

## Conclusion

High circulating GDF15 concentrations in ICU patients reflected severity of illness and poor prognosis and may contribute to aversive responses to enteral and oral nutrition. However, GDF15 could not identify patients who may benefit from or be harmed by early PN, and the association with enteral feeding (in)tolerance was weak. Hence, the potential of GDF15 as a “ready-to-feed indicator,” both from the viewpoint of metabolic tolerance for nutrition as well as gastrointestinal tolerance, appeared limited.

## Supplementary information

**Additional file 1.** **Table 1.** Inclusion and exclusion criteria of EPaNIC and DAS trials. Inclusion and exclusion criteria for patient recruitment in the EPaNIC and DAS trials.

**Additional file 2.** **Figure 1.** Caloric intake of early PN and late PN patients during the first week in ICU. Daily total caloric intake and caloric intake from enteral nutrition and parenteral nutrition are shown for the propensity score-matched subgroup of 564 early PN and 564 late PN patients during the first week in ICU (intervention window).

## Data Availability

Data sharing will be considered only on a collaborative basis with the principal investigators, after evaluation of the proposed study protocol and statistical analysis plan.

## Abbreviations

ANOVA: Analysis of variance
APACHE-II: Acute Physiology and Chronic Health Evaluation II
AUROC: Area under the receiver operating curve
BMI: Body mass index
CI: Confidence interval
CRP: C-reactive protein
EN: Enteral nutrition
HR: Hazard ratio
GDF15: Growth-differentiation factor-15
GI: Gastrointestinal
ICU: Intensive care unit
ISR: Integrated stress response
IQR: Interquartile range
MRC: Medical Research Council
NRS: Nutritional risk screening
OR: Odds ratio
PN: Parenteral nutrition
RCT: Randomized controlled trial
