# Supplementary material for: The clinical potential of GDF15 as a “ready-to-feed indicator” for critically ill adults
Source: Crit Care. 2020 Sep 14;24:557. doi: 10.1186/s13054-020-03254-1 (PMC7488998; doi:10.1186/s13054-020-03254-1)
Supplement: Supplementary file 1 — Additional file 1. Table 1. Inclusion and exclusion criteria of EPaNIC and DAS trials. Inclusion and exclusion criteria for patient recruitment in the EPaNIC and DAS trials. [file 13054_2020_3254_MOESM1_ESM.docx]

**Additional file 1. Table 1: Inclusion and exclusion criteria of EPaNIC and DAS trials**

| **EPaNIC trial: randomization upon ICU admission** | |
| --- | --- |
| **Inclusion criteria** | **Exclusion criteria** |
| Age ≥ 18 years | Moribund or coded DNR |
| NRS score ≥ 3 | Enrollment in another trial |
|  | Short-bowel syndrome |
|  | Home ventilation |
|  | Diabetic coma |
|  | Referred with nutritional regimen |
|  | Pregnant or lactating |
|  | No central catheter |
|  | Taking oral nutrition |
|  | Readmission to the ICU |
|  | BMI < 17 |
|  | No consent |
|  |  |
| **DAS trial: inclusion on day 7 in ICU** | |
| **Inclusion criteria** | **Exclusion criteria** |
| 7 days in ICU Age ≥ 18 years | Treatment with systemic glucocorticoids, etomidate, azoles, or other drugs predisposing to adrenal insufficiency |
|  | No vital organ support (defined as dependency on mechanical ventilation, and mechanical and/or pharmacological hemodynamic support) |
|  | No arterial or central venous catheter |
|  | Referral from another ICU |
|  | Cerebral/pituitary/adrenal disorders with impact on the neuroendocrine system |
|  | Enrollment in another trial |
|  | Expected death within 12 hours |
|  | No consent |

The inclusion and exclusion criteria for both the EPaNIC and DAS trials are listed.

Abbreviations: ICU: intensive care unit, NRS: nutritional risk screening, DNR: do not resuscitate, BMI: body mass index.
