# Supplementary material for: The clinical potential of GDF15 as a “ready-to-feed indicator” for critically ill adults
Source: Crit Care. 2020 Sep 14;24:557. doi: 10.1186/s13054-020-03254-1 (PMC7488998; doi:10.1186/s13054-020-03254-1)

**Additional file 2. Figure 1: Caloric intake of Early-PN and Late-PN patients during the first week in ICU**

Daily total caloric intake and caloric intake from enteral nutrition and parenteral nutrition are shown for the propensity score-matched subgroup of 564 Early-PN (shown in black) and 564 Late-PN (shown in grey) patients during the first week in ICU (intervention window). Diamonds indicate medians, and whiskers represent interquartile ranges. Numbers below the graphs indicate the number of patients who were still in the ICU on the respective days.


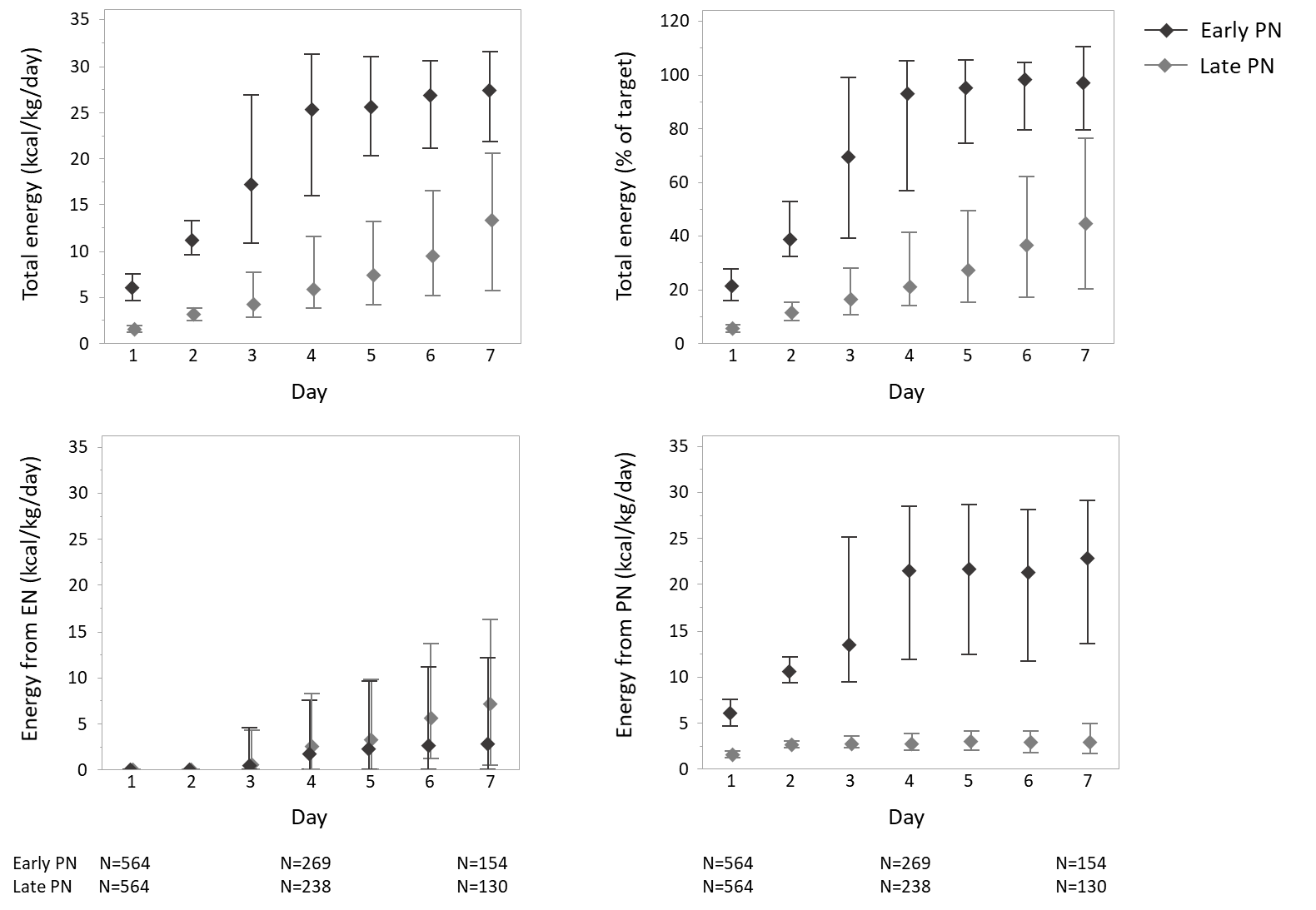

Supplement: Supplementary file 2 — Additional file 2. Figure 1. Caloric intake of early PN and late PN patients during the first week in ICU. Daily total caloric intake and caloric intake from enteral nutrition and parenteral nutrition are shown for the propensity score-matched subgroup of 564 early PN and 564 late PN patients during the first week in ICU (intervention window). [file 13054_2020_3254_MOESM2_ESM.docx]
